# Factors Associated With Knowledge of Sickle Cell Disease and Haemoglobin Genotype Awareness Among Pregnant Women in Three Health Centres in Libreville and Owendo, Gabon

**DOI:** 10.1155/anem/7713981

**Published:** 2026-10-04

**Authors:** Marielle Igala, Rick Farel Ewendje, Nathalie Ambounda Ledaga, Léonie Esther Ledaga Lentombo, Paule Marcelle Bamba Moussounda, Graziella Dolores Helley Ondo, Sarah Ikhogou Mensah, Axel Dany Ollo Ebozogo, Jean Bruno Boguikouma

**Affiliations:** ^1^ Department of Medicine University of Health Sciences Libreville Gabon akdeniz.edu.tr; ^2^ Internal Medicine Service Libreville University Hospital Center Libreville Gabon; ^3^ Department of Gynecology University of Health Sciences Libreville Gabon akdeniz.edu.tr; ^4^ Department of Gynecology and Obstetrics Libreville University Hospital Center Libreville Gabon

**Keywords:** Gabon, haemoglobin genotype, knowledge, pregnant women, sickle cell disease

## Abstract

**Introduction:**

In Gabon, screening for sickle cell disease, a hereditary disorder that causes haemolytic anaemia due to the sickling of red blood cells, is systematic during pregnancy and mandatory before marriage. Several years after the implementation of these measures, this study aimed to identify the factors associated with knowledge of sickle cell disease and awareness of haemoglobin genotype among pregnant women.

**Patients and Methods:**

This was a multicentre, cross‐sectional, descriptive and analytical study conducted from May to December 2024 in three health centres in Greater Libreville. Pregnant women of any age, regardless of gestational age, who agreed to participate by giving written consent were included. Statistical analysis was performed using Epi‐Info software, version 7.2.3.1. Multivariable logistic regression was additionally performed to identify independent determinants of haemoglobin genotype awareness and of general knowledge of sickle cell disease.

**Results:**

A total of 300 women with a mean age of 26.6 ± 5.9 years were included. They were mostly single (56%), in training or apprenticeship (42%), with a secondary education level (54%). Haemoglobin genotype was known to 128 (42.7%) participants. Awareness of one’s own genotype was independently associated with age (aOR = 1.12, 95% CI 1.07–1.18), number of antenatal visits (aOR = 1.46, 95% CI 1.26–1.69), and tertiary education (aOR = 5.61, 95% CI 1.66–19.02), whereas marital status, significant in bivariate analysis, was not an independent determinant after adjustment.

**Conclusion:**

Mandatory screening policies alone are insufficient to ensure adequate awareness of sickle cell disease and haemoglobin genotype. Antenatal care visits represent a concrete, currently underused opportunity for structured counselling; integrating progressive, education‐adapted genetic counselling into routine antenatal care should be prioritised to strengthen the public health impact of Gabon’s existing screening programmes.

## 1. Introduction

Sickle cell disease (SCD) is the most common inherited haemoglobin disorder worldwide. Each year, approximately 300,000 children are born with SCD globally, 85% of them in Africa [1]. The prevalence of the sickle cell trait (SCT) gene across the continent ranges from 10% to 40% depending on the country. The genetic basis of SCD transmission, together with its clinical severity and social consequences, has led many countries to implement preventive screening policies. In the Gabonese population, approximately 21% of individuals carry the SCT and 1.7% have SCD [2]. Screening for SCD/SCT is mandatory in two circumstances in Gabon: during antenatal care and before civil marriage, in accordance with Article 220 of Law No. 009/2010, which stipulates that no civil registrar may officiate a marriage without a medical certificate confirming that haemoglobin electrophoresis has been performed. Haemoglobin electrophoresis is also routinely prescribed during pregnancy monitoring, independent of this legal requirement.

Several years after the introduction of these mandatory screening measures, no data are available on whether they have translated into adequate knowledge of SCD and awareness of personal haemoglobin genotype among pregnant women in Gabon. Screening availability alone does not guarantee that patients understand their results or their implications; without effective counselling and communication of test results, mandatory testing policies may fail to achieve their intended public health impact. Identifying the factors that influence knowledge of SCD and awareness of haemoglobin genotype among pregnant women is therefore essential to strengthen counselling strategies and improve the effectiveness of existing national prevention programmes.

We hypothesised that sociodemographic characteristics and utilisation of antenatal care influence pregnant women’s knowledge of SCD and their awareness of their own haemoglobin genotype.

This study aimed to determine the level of knowledge of SCD and the frequency of awareness of haemoglobin genotype among pregnant women attending prenatal consultations at three maternal and child health centres in Greater Libreville and to identify the factors associated with this knowledge.

## 2. Methodology

This was a multicentre, cross‐sectional study with prospective and consecutive enrolment, conducted from May to December 2024 in three maternal and child health centres in Greater Libreville: La Peyrie, Glass and Louis. Pregnant women of any age and any gestational age who provided written informed consent were eligible for inclusion. Women who declined to participate and those who had received a blood transfusion within the previous 3 months were excluded.

Haemoglobin genotype screening using the HemoTypeSC test was performed as part of routine antenatal care, in accordance with Gabon’s mandatory prenatal screening policy for SCD (Article 220, Law No. 009/2010); no additional biological sampling was performed specifically for research purposes beyond this legally mandated screening. Institutional authorisation for the study, including administration of the knowledge questionnaire, was obtained from each of the three participating health centres, and written informed consent was obtained from all participants prior to enrolment, in accordance with the Declaration of Helsinki.

The minimum required sample size was calculated using the formula *n* = *z* ^2^ *p*(1 − *p*)/*d* ^2^, where *p* is the estimated prevalence of SCT in Gabon (21%) [2], *z* is the value corresponding to a 95% confidence level (1.96), and *d* is the acceptable margin of error (5%), yielding a minimum requirement of 254 women.

Data were collected using a structured, interviewer‐administered questionnaire written in French, developed specifically for this study—as no validated instrument assessing knowledge of SCD and haemoglobin genotype awareness among pregnant women is available in the Gabonese context—and pretested on a small sample of 10 pregnant women prior to the main survey. The questionnaire comprised 20 items across three sections: (i) sociodemographic characteristics (age, marital status, occupation, residence, nationality); (ii) obstetric history (gestational age, number of antenatal visits, gravidity, parity) and (iii) awareness of personal and partner haemoglobin genotype, family or social history of SCD, sources of information on SCD, and six knowledge‐assessment items.

Each of the six knowledge items was scored one point if answered correctly, yielding a total knowledge score ranging from 0 to 6, categorised by the investigators as poor (0–2), medium (3–4) or good (5–6), as reported in Table 1. Haemoglobin genotype was determined using the HemoTypeSC test (Silver Lake Research Corporation), a point‐of‐care qualitative immunochromatographic assay identifying haemoglobin A, S and C, selected for its reliability, rapid turnaround and ease of use in field conditions.

**TABLE 1:** Assessment of participants’ knowledge of sickle cell disease: item‐level responses and overall knowledge score.

Question	Correct answer, *n* (%)	Don’t know, *n* (%)	Incorrect answer, *n* (%)	Total
*(a) Distribution of responses to knowledge questions*				
Transmission pathway of SCD	188 (62.7)	96 (32.0)	16 (5.3)	300 (100)
Anomaly causing SCD	37 (12.3)	137 (45.3)	127 (42.4)	300 (100)
Disease clinical signs	153 (51.0)	99 (33.0)	48 (16.0)	300 (100)
Diagnosis (electrophoresis)	180 (60.7)	114 (38.0)	4 (1.3)	300 (100)
SCD treatment	22 (7.3)	278 (92.7)	—	300 (100)
Pregnancy‐related risks	32 (10.7)	268 (89.3)	—	300 (100)

**Level**	**N**		**%**	

*(b) Distribution by overall knowledge level*				
Poor [0–2]	147		49.0	
Medium [3–4]	148		49.3	
Good [5–6]	5		1.7	
Total	300		100	

Item‐level coding underlying the investigators’ original knowledge score were not separately retained in the analytic dataset. For the purposes of multivariable analysis, we therefore re‐derived a structured six‐item knowledge score directly from item‐level free‐text responses, using a pre‐specified, clinically justified correct‐answer coding grid (described in detail in the Limitations section). This structured score was categorised as low (≤ 2), moderate (3–4), or good (≥ 5). For the second multivariable model, this structured score was dichotomised as moderate‐to‐good (≥ 3/6) versus low (≤ 2/6).

Data was recorded anonymously in Excel 2016 (Microsoft Corp.) and analysed using Epi Info software, Version 7.2.3.1 (CDC, Atlanta, USA). Qualitative variables were expressed as frequencies and percentages, and quantitative variables as means with standard deviations. Associations between qualitative variables were assessed using the chi‐squared test or Fisher’s exact test where appropriate. To identify independent determinants of haemoglobin genotype awareness and of general SCD knowledge, multivariable logistic regression was additionally performed using Python (statsmodels), with adjusted odds ratios (aORs) and 95% confidence intervals reported; multicollinearity was assessed using variance inflation factors. Statistical significance was set at *p* < 0.05.

Candidate covariates for multivariable logistic regression (age, marital status, education level, nationality and number of antenatal visits) were selected a priori based on variables significantly associated with the outcomes in bivariate analysis (*p* < 0.05) together with age, retained as a covariate of established biological relevance; nationality, not examined in the original bivariate analysis, was retained a priori given its relevance to Gabon’s screening policy. Gravidity, parity and gestational age were excluded from the final models because of collinearity with age and number of antenatal visits (variance inflation factor > 5 in preliminary models).

## 3. Results

A total of 300 women were enrolled. The mean age of participants was 26.6 ± 5.9 years (range 14–43). The majority (65.7%) were between 18 and 29 years old (Table 2).

**TABLE 2:** Participants’ sociodemographic characteristics.

Variable	*n*	%
Health centre		
La Peyrie	119	39.7
Glass	138	46.0
Louis	43	14.3
Total	300	100
Municipality of residence		
Libreville	253	84.0
Owendo	33	11.0
Akanda	14	5.0
Marital status		
Single	166	56.0
Live‐in partner	82	27.0
Married	42	14.0
Engaged	10	3.0
Level of education		
Secondary	163	54.0
Tertiary	110	37.0
Primary	27	9.0
Occupation		
Formation/apprenticeship	126	42.0
Unemployed	84	28.0
Seller	43	14.3
Manufacturer	18	6.0
Health professional	14	4.7
Public servant	12	4.0
Law enforcement officer	3	1.0

The women had a mean gestational age of 21.1 ± 8.3 weeks of amenorrhoea (range 6–39). Half of the participants had attended at least two antenatal consultations at the time of the survey. Mean gravidity was 2.2 ± 1.4 (range 0–7), and mean parity was 0.9 ± 1.3 (range 0–6). An analysis of the participants’ awareness of haemoglobin genotype revealed that just under half (128, 42.6%) reported knowing their genotype (Table 3). Among those who knew their haemoglobin genotype, 30.3% were Hb AA, 12% Hb AS and 0.3% Hb AC. Two hundred fifty‐three women (84.3%) did not know their spouse’s haemoglobin genotype; when known, the profile was most often normal (12.3% Hb AA and 3.3% Hb AS).

**TABLE 3:** Haemoglobin genotype awareness and sickle cell disease information sources.

Variable	*n*	%
Haemoglobin genotype awareness—pregnant woman		
Known	128	42.7
Unknown	172	57.3
Haemoglobin genotype awareness—spouse		
Known	47	15.7
Unknown	253	84.3
Spouse’s genotype awareness (married women only)		
Known	11	26.2
Unknown	31	73.8
Family history/known acquaintance with SCD		
No known case	212	70.7
Collateral relative	48	16.0
Non‐family acquaintance (friend/neighbour)	34	11.3
Ascendant (mother/grandmother)	4	1.3
Descendant (child)	2	0.7
Sickle cell disease information sources		
Hospital and health personnel	115	38.3
School	63	21.0
Family	39	13.0
News/media	39	13.0
Sensitisation campaign	7	2.3
No information	37	12.3

Overall, 78.3% of participants reported not having sufficient information about SCD, versus 9.3% who considered their knowledge good and 12.4% unable to say. The detailed responses are shown in Table 1. Only 1.7% had a knowledge level considered good overall (Table 1).

The SCT test revealed that 69 women (23%) were carriers of the SCT (AS).

There was no association with gravidity or parity. A significant bivariate association was observed between haemoglobin genotype awareness and marital status (*p* = 0.046): 12 of the 42 married women (28.6%) knew their genotype, compared with 116 of the 259 single women (44.8%). However, as detailed below in the multivariable analysis, this association did not persist after adjustment for age.

There was a significant association between genotype awareness and gestational age at the time of consultation/survey (*p* = 0.001): women earlier in pregnancy (mean ∼19 weeks) were significantly less informed than those further along (mean ∼23 weeks). Genotype awareness was also significantly associated with the number of antenatal visits (*p* = 0.001).

A statistical association was found between overall knowledge level and marital status, level of education, health centre attended and number of procedures (all *p* < 0.05) (Table 4).

**TABLE 4:** Relationship between knowledge level and age, marital status, education level, residence, and health center.

Variable	Good	Medium	Poor	Total	*p*
Age					0.749
< 18	0	4	4	8	
18–29	4	101	92	197	
30–45	1	43	51	95	
Total	5	148	147	300	
Marital status					**0.047**
Single	0	81	85	166	
Live‐in partner	2	47	33	82	
Married	1	17	24	42	
Engaged	2	3	5	10	
Education level					**0.0001**
Primary	0	11	16	27	
Secondary	0	98	65	163	
Tertiary	5	39	66	110	
Municipality of residence					**0.15**
Libreville	5	119	129	253	
Owendo	0	23	10	33	
Akanda	0	6	8	14	
Health centre					**0.001**
La Peyrie	2	29	88	119	
Glass	2	110	26	138	
Louis	1	9	33	43	
Gravidity					**0.0129**
1 pregnancy	4	51	41	96	
2 pregnancies	1	51	55	107	
> 3 pregnancies	0	46	51	97	

*Note:* Bold values represent the statistical relation between knowledge and epidemiological parameter. It means that there is a link between parameters.

No statistically significant association was observed between gravidity and knowledge level nor between haemoglobin genotype and choice of spouse (Table 4).

## 4. Multivariable Determinants of Haemoglobin Genotype Awareness and General Knowledge

To further investigate the determinants of haemoglobin genotype awareness and of general SCD knowledge, two multivariable logistic regression models were fitted (*n* = 296, after exclusion of cases with missing covariate data). Genotype awareness rose steadily with antenatal care contact: only 28.2% of women with a single antenatal visit knew their genotype, compared with 39.8% among those with two to three visits and 69.2% among those with four or more visits, a nearly 2.5‐fold increase between the lowest and highest visit categories. A similar trend was observed by education level, from 22.2% among women with primary education to 30.2% with secondary and 65.4% with tertiary education: women with tertiary education were nearly three times as likely as those with primary education to know their genotype (Figure 1A). Awareness also differed by nationality, known to 47.3% of Gabonese women versus 15.9% of non‐Gabonese women, Gabonese women being nearly three times as likely as non‐Gabonese women to be aware of their genotype. After adjustment for these factors, marital status was no longer associated with awareness (Figure 1A).

**Figure 1:**
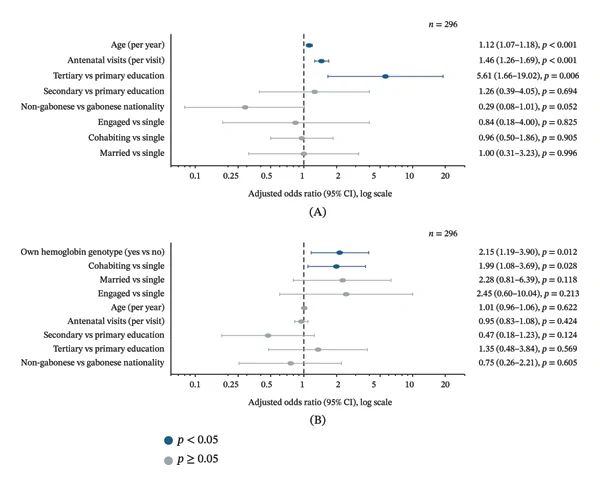
Determinants of haemoglobin genotype awareness (A) and of adequate general knowledge of sickle cell disease (B) among pregnant women, multivariable logistic regression.

Moreover, adequate general knowledge of SCD was almost twice as frequent among women who already knew their own genotype (50.0%) than among those who did not (27.3%) and about 1.5 times more frequent among women in a cohabiting relationship (46.3%) than among single women (30.7%). Age, education, nationality and antenatal visit frequency showed no comparable association for this outcome (Figure 1B).

## 5. Discussion

In this study, the mean age of participants was 26.6 ± 5.9 years, with the largest group aged 18–29 years. This is consistent with Igala et al. [3], who reported a similar mean age (25.5 ± 5.4 years) among pregnant women with SCD in Libreville, and with Ezenwosu et al. [4] in Nigeria (20–29 years), though slightly younger than the 26–35‐year range reported by Tutuba et al. [5] in Tanzania. This age distribution reflects the period of peak fertility in these settings, with variation likely attributable to cultural and religious differences in age at first pregnancy across countries (see Tables 5 and 6).

**TABLE 5:** Relationship between electrophoretic profile knowledge and marital situation.

Electrophoretic profile knowledge	Married	Single	Total	*p*
				**0.046**
Know her profile	12	116	128	
Don’t know her profile	30	142	172	
Total	42	259	300	

*Note:* The bold value represents the statistical link between electrophoretic profile knowledge and marital situation.

**TABLE 6:** Relationship between the knowledge level and age, marital situation, education level, residence municipality and the attended health centre.

Variables	Good	Medium	Poor	Total	*p*
Age					0.749
< 18	00	04	04	08	
18–29	04	101	92	148	
30–45	01	43	51	147	
Total	05	148	147	300	
Marital situation					**0.047**
Single	00	81	85	166	
Live‐in partner	02	47	33	82	
Married	01	17	24	42	
Engaged	02	03	05	10	
Total	05	148	147	300	
Education level					**0.0001**
Primary	00	11	16	27	
Secondary	00	98	65	163	
Tertiary	05	39	66	110	
Total	05	148	147	300	
Municipality of residence					0.15
Libreville	05	119	129	253	
Owendo	00	23	10	33	
Akanda	00	06	08	14	
Total	05	148	147	300	
Health centre					**0.001**
La Peyrie	02	29	88	119	
Glass	02	110	26	138	
Louis	01	09	33	43	
Total	05	148	147	300	
Pregnancy number					**0.0129**
Pregnancy = 1	04	51	41	96	
Pregnancy = 2	01	51	55	107	
Pregnancy > 3	00	46	51	97	
Total	05	148	147	300	

*Note:* Bold values represent the statistical link between epidemiological parameter and knowledge.

Just over half of participants (57.3%) were unaware of their own haemoglobin genotype despite mandatory antenatal screening. This finding suggests that legal and institutional screening requirements alone may be insufficient to ensure adequate awareness if they are not accompanied by effective counselling and communication of results. The multivariable analysis is consistent with this interpretation: awareness of haemoglobin genotype increased independently with age (aOR = 1.12, 95% CI 1.07–1.18) and, most notably, with the number of antenatal care visits (aOR = 1.46, 95% CI 1.26–1.69), suggesting that repeated contact with the health system is associated with greater awareness beyond the screening event itself. This is in accordance with the observation that women earlier in pregnancy were significantly less informed than those further along (*p* < 0.001) and supports the possibility that awareness may increase progressively across successive antenatal visits, rather than resulting from a single screening event. Comparable gaps have been reported elsewhere in the region: Ezenwosu et al. found that only 24.7% of pregnant women in Nigeria were unaware of their status, considerably lower than our figure, which may reflect differences in how screening results are communicated across settings rather than differences in screening coverage itself [4].

Tertiary education was independently associated with awareness of haemoglobin genotype (aOR = 5.61, 95% CI 1.66–19.02), whereas secondary education was not distinguishable from primary education in the adjusted model. These findings suggest that higher educational attainment is associated with greater awareness of haemoglobin genotype; whether this reflects differences in health literacy or in the ability to understand counselling cannot be determined from this cross‐sectional study. Simplified, visual and orally delivered information may nonetheless be worth considering for women with primary or secondary education, rather than relying on written material alone. This finding is consistent with reports from Togo, Nigeria and Ghana, where educational attainment has similarly been associated with SCD‐related knowledge or favourable attitudes towards screening and counselling [6–8].

Notably, marital status, which was significantly associated with awareness of haemoglobin genotype in bivariate analysis (*p* = 0.046), lost statistical significance after adjustment for age, education, nationality and antenatal care attendance. This suggests that the association observed in the unadjusted analysis was largely explained by age, since married women in our sample were on average older, and age was itself an independent predictor of awareness. This distinction matters clinically, therefore, as marital status is not, in itself, a barrier or facilitator of awareness, and counselling strategies should not be differentiated by marital status but rather target younger, less‐educated women regardless of relationship status.

Non‐Gabonese nationality was associated with a trend towards lower awareness of haemoglobin genotype, close to the threshold of significance (aOR = 0.29, 95% CI 0.08–1.01, *p* = 0.052). Although this association did not reach conventional significance, it may reflect differences in exposure to Gabon’s screening programmes or other unmeasured social, linguistic or administrative factors. This warrants confirmation in a larger sample but is, to our knowledge, a novel observation in this context.

Regarding general knowledge of SCD (beyond awareness of one’s own genotype), the mean knowledge score was low (1.89 ± 1.46 out of 6), with 63% of participants classified as having poor knowledge. This is considerably lower than the level of knowledge participants attributed to themselves before the survey, since 79.3% initially considered themselves well informed. In multivariable analysis, general knowledge was independently associated with cohabiting relationship status (aOR = 1.99, 95% CI 1.08–3.69) and, strongly, with awareness of one’s own haemoglobin genotype (aOR = 2.15, 95% CI 1.19–3.90), but not with age, education, nationality or antenatal care attendance. This divergence between the determinants of general knowledge and those of genotype awareness suggests that these two dimensions of knowledge may reflect partly distinct underlying mechanisms. The cross‐sectional design does not allow us to establish the direction of the association between awareness of one’s own status and general knowledge, being tested and informed of one’s result may itself be an opportunity for broader education, or, conversely, women with greater baseline knowledge may be more likely to seek and retain their result. Longitudinal studies are needed to disentangle this relationship. The explanatory power of this second model was modest (pseudo‐*R* ^2^ = 0.099), indicating that other unmeasured factors, such as the quality of counselling received, health literacy, or specific content of information sources, likely play a substantial role and were not captured by the variables available in this study.

Analysis of individual knowledge items revealed marked heterogeneity: while transmission (62.7% correct) and the diagnostic test (60.7% correct) were relatively well known, awareness of treatment modalities (7.3%) and pregnancy‐related risks (10.7%) were strikingly low. Existing information sources, most commonly hospital and healthcare staff (38.3%), appear to provide basic information on SCD but may insufficiently address its practical management or its specific implications for pregnancy, precisely the information most relevant to antenatal counselling [9].

Fifty‐one percent of participants correctly identified physical signs associated with SCD (jaundice, pallor and growth retardation), a proportion comparable to the 61% reported in Cameroon [10]. It is important to note that such phenotypic signs are only informative for individuals with SCD; SCT carriers, who account for the majority of carriers in the Gabonese population, are typically asymptomatic and cannot be identified by physical examination. Reliance on visible signs to ‘recognise’ carriers may therefore contribute to misconceptions regarding carrier status, such as a mistaken belief that the absence of visible signs excludes carrier status, a misconception with direct implications for premarital and reproductive counselling that, to our knowledge, has not been previously highlighted in this population.

A striking finding was that 84.3% of participants did not know their partner’s haemoglobin genotype, and partner choice was not associated with the partner’s genotype (*p* > 0.05), a proportion similar to the 75% reported by Nana Njamen et al. [10]. Thus, the prenuptial screening requirement, while legally enforced, does not appear to be accompanied by adequate communication of partner genotype or informed reproductive counselling, underscoring the need for structured couple‐based counselling rather than administrative certification alone.

The frequency of SCT observed in this population (23%) was consistent with the national estimate of 21% [2]. No haemoglobin C carriers were identified, despite just over 10% of participants originating from the western regions of the continent where this variant is more common; the increasing mobility of populations across the region nonetheless justifies continued systematic screening for this variant.

Taken together, these findings indicate that mandatory screening policies, in their current implementation, may not be sufficient on their own to ensure adequate awareness and understanding of SCD among pregnant women. Structured genetic counselling delivered progressively across antenatal visits, adapted to educational level, and extended to partners, may help translate legal screening requirements into greater public health impact. Qualitative findings demonstrate that young adults generally hold positive perceptions towards genetic counselling for SCD. Even though awareness of where such counselling can be accessed remains limited, these findings reinforce the case for embedding structured counselling within existing antenatal care services rather than relying on screening alone. This is also consistent with recommendations from the Democratic Republic of Congo, where strong demand for structured genetic counselling existed among mothers of children with SCD [11].

## 6. Limitations of the Study

This study has some limitations. First, participants were recruited from three health centres in a single urban area (Greater Libreville), which limits the generalisability of these findings to rural populations, where access to antenatal care and health information may differ substantially; future multicentre studies including rural settings and longitudinal follow‐up are needed to assess changes in knowledge over time, particularly following educational interventions. Second, the cross‐sectional design does not allow causal inference, and in particular cannot establish the direction of the association observed between awareness of haemoglobin genotype and general SCD knowledge. Third, knowledge and awareness were assessed using self‐reported responses to an interviewer‐administered questionnaire, which may be subject to recall and social desirability bias. Fourth, the six‐item knowledge score used in this study was developed specifically for this study and has not been formally validated against an external reference instrument; item‐level correlation coefficients and psychometric properties should be established in future work before wider use.

## 7. Conclusion

Several years after the introduction of mandatory sickle cell screening during pregnancy and before marriage, awareness of haemoglobin genotype among pregnant women in Greater Libreville remains limited, with more than half unaware of their own status and the vast majority unaware of their partner’s. Awareness of one’s own genotype was independently associated with age, tertiary education and, most actionably, the number of antenatal care visits, while marital status was not an independent determinant once age was accounted for. These findings indicate that mandatory screening alone may not be sufficient to guarantee adequate awareness, and that repeated antenatal contacts may provide an important and currently underused opportunity for structured counselling. Integrating structured, progressive genetic counselling, extended to partners and adapted to educational level, into routine antenatal care should be prioritised to strengthen the public health impact of Gabon’s existing screening programmes.

## Author Contributions

Conceptualisation: Marielle Igala; methodology: Marielle Igala; formal analysis: Marielle Igala, Rick Farel Ewendje; investigation: Marielle Igala, Rick Farel Ewendje, Nathalie Ambounda Ledaga, Léonie Esther Ledaga Lentombo, Paule Marcelle Bamba Moussounda, Graziella Dolores Helley Ondo, Sarah Ikhogou Mensah, Axel Dany Ollo Ebozogo; writing–original draft: Marielle Igala, Rick Farel Ewendje, Nathalie Ambounda Ledaga; writing–review and editing: Marielle Igala, Rick Farel Ewendje, Nathalie Ambounda Ledaga; supervision: Jean Bruno Boguikouma.

## Funding

This research did not receive specific funding.

## Disclosure

All authors have read and approved the final version of the manuscript. Marielle Igala, the corresponding author, had full access to all of the data in this study and takes complete responsibility for the integrity of the data and the accuracy of the data analysis.

## Ethics Statement

Haemoglobin genotype screening using the HemoTypeSC test was performed as part of routine antenatal care, in accordance with Gabon’s mandatory prenatal screening policy for sickle cell disease (Article 220, Law No. 009/2010); no additional biological sampling was performed specifically for research purposes beyond this legally mandated screening. Institutional authorisation for the study, including administration of the knowledge questionnaire, was obtained from each of the three participating health centres, and written informed consent was obtained from all participants prior to enrolment, in accordance with the Declaration of Helsinki.

## Conflicts of Interest

The authors declare no conflicts of interest.

## Data Availability

The raw data supporting the findings are included in the article and available from the corresponding author upon request.
